# Extracorporeal therapy procedures (plasma exchange and immunoadsorption) in chronic inflammatory demyelinating polyneuropathies (CIDP)– a database analysis

**DOI:** 10.1186/s12883-025-04294-2

**Published:** 2025-07-17

**Authors:** Moritz Mahlberg, Johannes Dorst, Zeynep Elmas, Ulrike Haase, Michael Heming, Louisa Müller-Miny, Gerd Meyer zu Hörste, Mark Stettner, Florian Then Bergh, Petra Baum, on behalf of the Disease-Related Competence Network Peripheral Nervous System (KKPNS)

**Affiliations:** 1 Department of Neurology, University of Leipzig, Leipzig, Germany; 2 Department of Neurology, University of Ulm, Ulm, Germany; 3 German Center for Neurodegenerative Diseases, Ulm, Germany; 4 Department of Neurology, Heinrich-Braun-Klinikum Zwickau, Zwickau, Germany; 5 Department of Neurology with Institute of Translational Neurology, Medical Faculty, University Hospital Münster, Münster, Germany; 6 Department of Neurology, Working Group for Clinical and Experimental Neuroimmunology, University Hospital Essen, Essen, Germany

**Keywords:** chronic inflammatory demyelinating polyneuropathy, neurological autoimmune diseases, immunoadsorption, plasma exchange, treatment, INCAT score, Hughes score

## Abstract

**Background and objectives:**

There is limited study data on both therapeutic plasma exchange (PE) and immunoadsorption (IA) in chronic inflammatory demyelinating polyneuropathy (CIDP), mostly based on case series in patients in the early stages of the disease.

The aim of this retrospective study was to compare the efficacy and tolerability of the two therapy procedures in a larger sample with a longer duration of disease and immunomodulatory pre-treatment.

**Methods:**

In this retrospective study from 5 centers in Germany, register data on the efficacy, safety and tolerability of therapy with IA or PE in patients with CIDP were examined. Treatment response was assessed using neurological scores (INCAT and Hughes Score), duration of hospital stay, and subjective assessment by examiners and patients. In addition, side effects were recorded.

**Results:**

A total of 44 patients were analyzed, 23 treated with PE (mean age 61.3 years, 17 male, 6 female) and 21 with IA (mean age 67, 14 male, 7 female).

The mean duration of disease before treatment was 8.48±3.82 years (PE group) and 7.24±3.15 years (IA group). IA and PE showed a comparable treatment response. With IA, 11 out of 21 (52.4%) patients improved, whereas with PE, 14 out of 23 (60.9%) patients improved. The differences between before- and after-treatment INCAT and Hughes scores also showed an improvement with both PE and IA individually (INCAT 1.17±1.61 and 0.71±1.65, respectively, Hughes score 0.48±0.73 and 0.33±0.66, respectively), while there were no significant differences between the two groups. The patients in the IA group had a significantly shorter inpatient stay (*p* < 0.019). There was one adverse event in each group, but no serious adverse events in either group.

**Discussion:**

This retrospective study indicates that IA and PE show comparable efficacy in chronic autoimmune neuropathies, including patients with longer disease duration and immunomodulatory pretreatment.

## Introduction

Immune-mediated or synonymously autoimmune neuropathies are rare disorders, with a prevalence of about 6–8 per 100,000, and characterized by a misguided immune response towards the peripheral nerve. They cause distinct combinations of sensory, motor and autonomic manifestations. A distinction is made between acute (primarily Guillain-Barré syndrome [GBS]) and chronic forms, the most common of which is chronic inflammatory demyelinating polyradiculoneuropathy (CIDP) and its variants [1]. Although there has been considerable progress in research into the pathogenetic mechanisms, the causes underlying these immune mediated neuropathies have not yet been conclusively clarified [2]. Complex mechanisms involving both humoral and cellular immunity are being indicated, and diverse therapies targeting the immune response are therefore applied [3, 4].

Key aims of treatment are to resolve and prevent the local inflammatory processes, resulting in improved nerve conduction and preservation or repair of the peripheral nerve myelin, in order to prevent secondary axonal degeneration which may lead to permanent disability [5].

Although research results based on comparative studies are low (very low safety), plasma exchange and IVIg appear to be equally effective for induction therapy in CIDP and PE is recommended in the European Academy of Neurology/Peripheral Nerve Society (EAN/PNS) guidelines [6]. However in guidelines, there are no recommendations for IA due to insufficient study data. Extracorporeal therapy, i.e. PE and IA, are used either alone or in combination with immunomodulatory compounds [7]. PE and IA have the common goal of eliminating pathogenic autoantibodies, involved in immune neuropathies, from the blood [8]. The therapeutic effect of these two methods can be attributed to three main mechanisms: (1) immediate reduction of the intravascular concentration of (auto)antibodies, (2) pulsed induction of antibody redistribution, and (3) additional immunomodulatory changes induced by this. Besides reducing (auto)antibodies, both apheresis techniques also modulate other blood proteins, e.g. some cytokines, chemokines and coagulation factors [7, 9]. Both therapy methods (PE and IA) require vascular access, often established by central venous catheter; complications can occur primarily associated with venous access, but these and other side effects generally occur rarely [5, 7–18].

Few studies to date have compared treatment with extracorporeal therapies [9, 19, 20]. These studies included only a small number of patients with autoimmune neuropathies [19], compared IA and PE in the early stage of CIDP [20] or reported on IA alone [9]; still, the results showed that extracorporeal therapies were effective and well tolerated, resulting in the inclusion of these methods into treatment guidelines [6, 21].

The aim of this retrospective study was to analyze the two extracorporeal therapy procedures, PE and IA, in the treatment of chronic immune neuropathies, extending the information provided in prior studies by using a larger, well-defined cohort of CIDP patients with a longer duration of disease and immunomodulatory pre-treatment.

## Methods and statistics

In this study, retrospective data from the KKPNS (Disease-Related Competence Network Peripheral Nervous System) database were used. This is a neurological research network and database that includes data from sites across Germany to study clinical aspects of immune neuropathies. Data acquired between 2018 and 2023 at the following locations were evaluated: Essen (10 patients), Münster (5 patients), Ulm (14 patients), Zwickau (7 patients) and Leipzig (8 patients; see Table 1). This study was approved by the local ethics committee (Approval Number Leipzig: 409/29-lk) and the entire study was conducted in accordance with the Declaration of Helsinki. Written informed consent was obtained by all participants. The inclusion criteria were as follows:

All Datasets with apheresis treatments in the mentioned centers and CIDP diagnosis according to EAN/PNS criteria [6] and at least one apheresis treatment series.

**Table 1:**
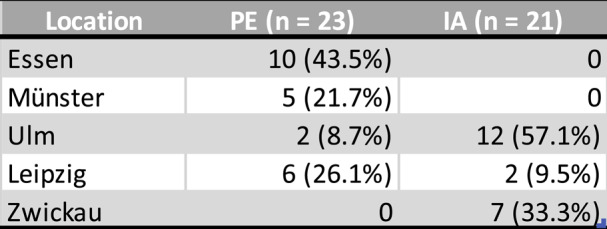
Locations

Table 1: Shows the number of patients at the locations and the distribution across therapy groups

The primary outcome variables were modified INCAT and Hughes scores measured before and after the index apheresis series [22]. The INCAT (Inflammatory Neuropathy Cause and Treatment) disability score and the Hughes Functional Grading Scale are standardized clinical tools used to assess functional impairment in patients with immune-mediated neuropathies. The INCAT score evaluates disability in both upper and lower limbs, particularly focusing on the ability to walk and perform manual tasks, while the Hughes scale provides a global assessment of motor disability ranging from normal function to complete paralysis. Both scores are widely used in clinical practice and research to monitor disease progression and treatment response [23–25]. The difference between the INCAT and Hughes scores before and after the apheresis series, side effects and the duration of inpatient treatment were included as additional variables.

In addition to INCAT and Hughes scale scores, additional clinician-rated outcome categories — namely, improvement, stability, and worsening — were recorded to allow integration of a more holistic evaluation of patient status, reflecting, e.g., nuanced changes in function.

The medium-term effect of the apheresis series was examined using secondary outcome variables (interval until the next apheresis, number of sessions per apheresis series and therapy success, measured after the index apheresis series by the subjective perception by patients and judgement of clinical examiners). Follow-up data were recorded as clinically indicated and feasible to the patients; no standardized intervals for follow-up visits were defined.

Side effects were recorded both by evaluating the data included in the KKPNS database and also retrospectively using the original medical records.

### Statistical methods

The study data were analyzed using descriptive statistical methods. To compare the two study groups with regard to their primary and secondary endpoints, the t-test for independent samples, the chi-square test and the non-parametric Mann-Whitney U test were used, as appropriate to the data structure, and as indicated. Correlations were examined using Spearman rank correlation analysis and a multiple regression analysis was performed. A *P*-value < 0.05 was considered statistically significant. All data are expressed as mean and standard deviation or as relative frequencies. IBM SPSS Statistics 29 was used for statistical analysis.

## Results

The data of a total of 44 patients (43 CIDP and 1 MADSAM), who underwent 44 series of extracorporeal treatment (23 with PE and 21 with IA), were evaluated.

Baseline characteristics are shown in Table 2. The PE and IA group were comparable in terms of age, sex, height, body weight, age at diagnosis and duration of the disease. The multiple regression analysis showed a significant influence of body weight (*p* < 0,05) and no significant influence of age, sex, height and duration of disease of the dependent variables.

**Table 2:**
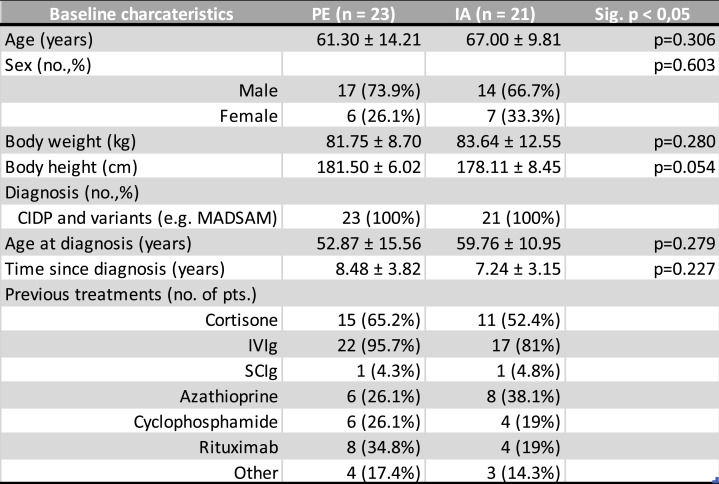
Baseline characteristics

Table 2: Presentation of the baseline characteristics of our study population, as shown for those in the PE (plasma exchange) and IA (immunoadsorption) groups, the values are given as mean ± standard deviation or as numbers with relative frequencies

Centers preferentially used either plasma exchange or immunoadsorption; the choice of the procedure was a result of practical, clinical, and center-specific factors. Of note, local availability played an important part in this decision; e.g., the Zwickau center performs immunoadsorption in their own Neurology department, easing its application. Furthermore, in several cases, IA was chosen based on individual patient considerations, particularly when there was a history of adverse reactions to prior therapies involving foreign protein administration, such as IVIG.

At the time of data collection (see Table 3), 10 patients (43.5%) in the PE group reported gait disturbance and 3 patients (13%) reported respiratory restriction due to CIDP or proximal paresis. In the IA group, the figures were 12 (57.1%) and 6 (28.6%), respectively. Thus, the IA group was more severely impaired. Sensory symptoms were similarly prevalent in both groups and present in most patients (22 patients [95.7%] in the PE group and 21 patients [100%] in the IA group). The majority of both groups had impairments in the lower or both lower and upper extremities. Involvement of the cranial nerves was more common in the PE group (5 patients (21.6%)) than in the IA group (3 patients (14.4%). In the PE group, 3 patients (13%) had oculomotor palsy, and 2 patients (8.6%) had facial palsy; in the IA group, there was one patient (4.8%) with oculomotor palsy and 2 patients (9.6%) with facial palsy. Dysarthria and dysphagia were observed in 3 patients (13%) each in the PE group; in the IA group, there was one patient (4.8%) with dysarthria and 2 patients (9.5%) with dysphagia.

**Table 3:**
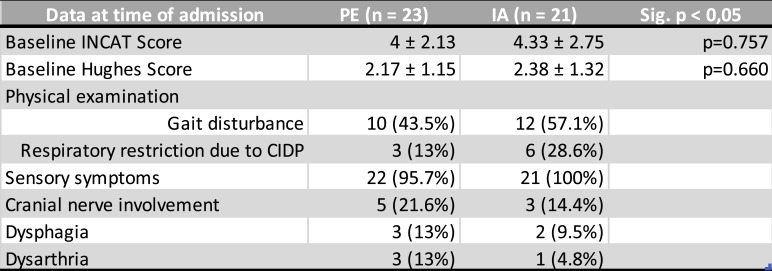
Clinical data before extracorporeal therapy

Table 3: Presentation of INCAT and Hughes score and clinical appearance before PE (plasma exchange) or IA (immunoadsorption), the values are given as mean ± standard deviation or as numbers with relative frequencies

### Apheresis-related results

**Fig. 1:**
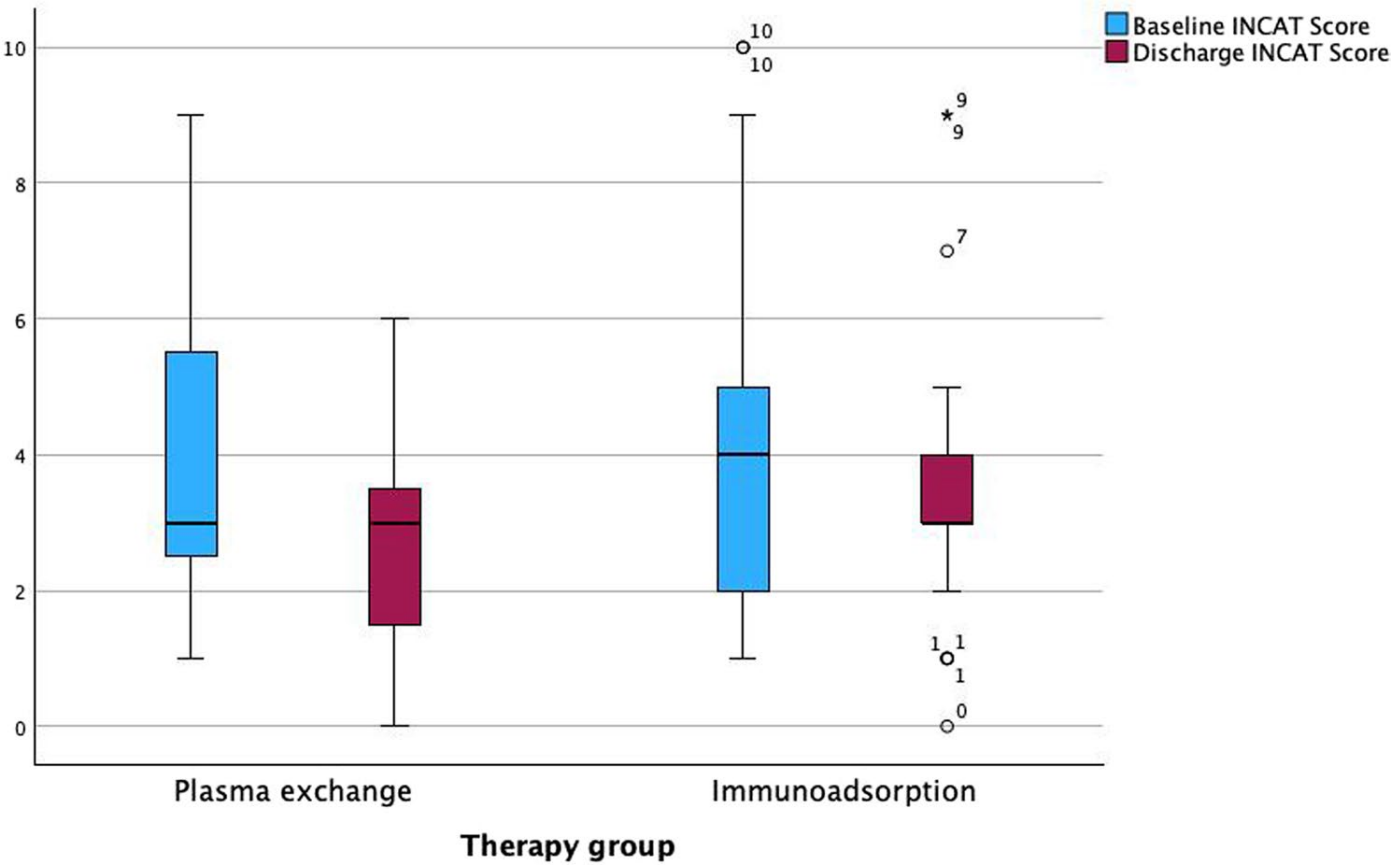
Baseline and discharge INCAT score. Presentation of baseline and discharge INCAT Score in both therapy groups

**Fig. 2:**
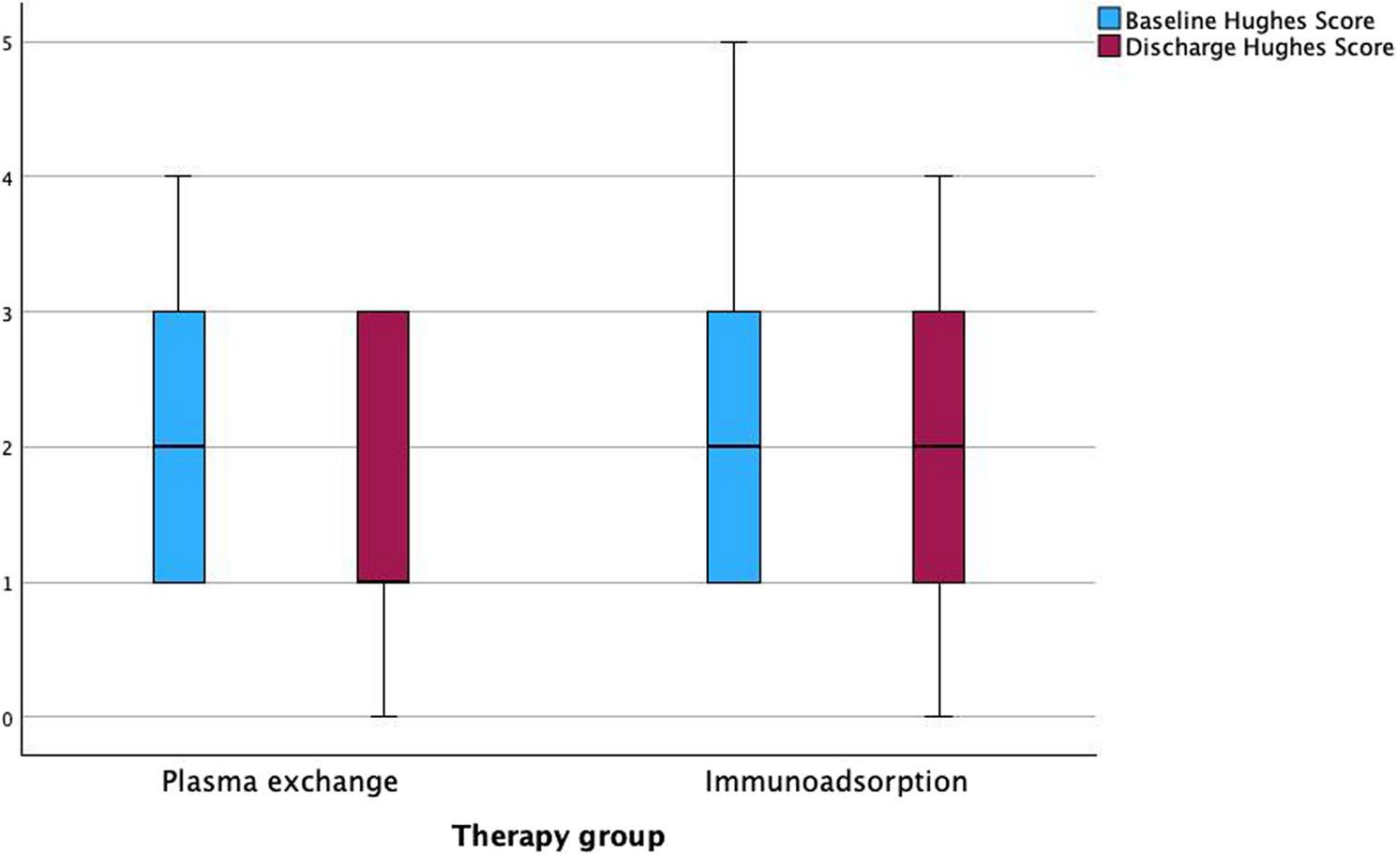
Baseline and discharge Hughes score. Presentation of baseline and discharge Hughes score in both therapy groups

Apheresis-related results are shown in Table 4. The baseline INCAT score of the PE group was 4.00±2.13 and that of the IA group 4.33±2.75. The baseline Hughes score in the PE group was 2.17±1.15 and in the IA group 2.38±1.32 (see Figs. 1 and 2). Neither baseline scores differed significantly between the two groups.

**Table 4:**
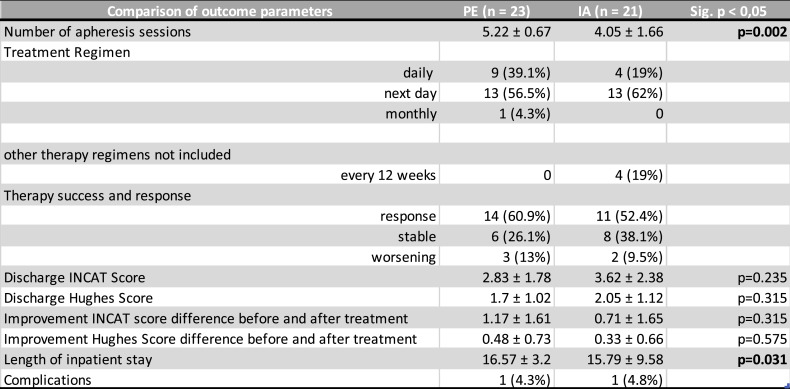
Comparison of outcome parameters

Table 4: Characterization of extracorporeal therapies and clinical outcomes in IA versus PE groups, length of stay was significantly different between the treatment groups (Wilcoxon-Mann-Whitney-Test); values are given as mean ± standard deviation or as a number with relative frequencies

The average number of apheresis sessions in the plasmapheresis group was 5.22 (±0.67) sessions and in the immunoadsorption group 4.05 (±1.66). This represents a significant difference. Both apheresis techniques were performed via central venous catheter or peripheral access. All participating centers used central venous access for the procedures. However, two patients from the Leipzig center were treated using peripheral venous access: one patient in the IA group and one in the PE group. The overall clinical condition was “improved” in over half (60.9%, 14 patients) in the PE group, “stable” in 6 patients (26.1%), and only 3 patients (13%) “deteriorated”. Similarly, in the IA group, 52.4% (11 patients) “improved”, one third were “stable” (38.1%), and 2 patients (9.5%) “deteriorated”. These judgements were a combination of the subjective perception of patients and clinical examination by examiners.

**Fig. 3:**
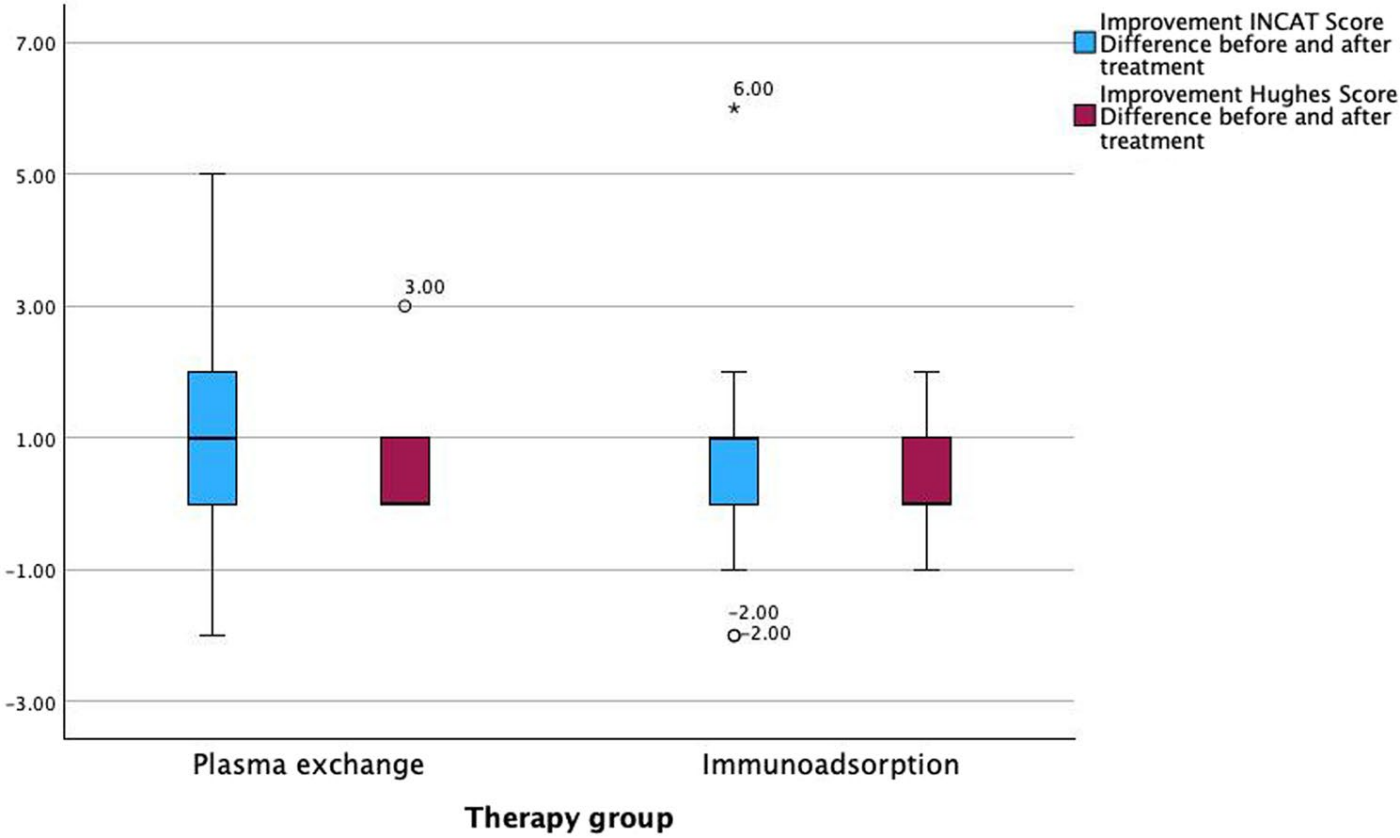
Improvement INCAT and Hughes score differences. Presentation of improvement INCAT and Hughes score differences in both therapy groups before and after treatment

Both treatment groups (PE and IA) showed an improvement in both the INCAT and the Hughes scores (INCAT to 2.83±1.78 and 3.62±2.38, respectively, Hughes score to 1.7±1.02 and 2.05±1.12, respectively) (see Figs. 1 and 2). The difference between before and after treatment in the INCAT score and the Hughes score also showed an improvement in PE and IA individually (INCAT 1.17±1.61 and 0.71±1.65, respectively, Hughes score 0.48±0.73 and 0.33±0.66, respectively) (see Fig. 3). Thus, there was no significant difference between the two groups. The length of stay in hospital during the procedures was 16.57±3.20 days in the PE group, and 15.79±9.58 days in the IA group (*p* < 0.05).

The therapy series was completed in all patients in the PE group (100%), and in 17 patients in the IA group (81%). In the PE group, 9 patients (39.1%) received treatment daily, 13 patients (56.5%) every next day, and 1 patient (4.3%) monthly. In the IA group, 4 patients (19%) received daily treatment, while 13 patients (62%) were treated every next day. At the time of data collection, the remaining 4 patients in the IA group received maintenance immunoadsorption at fixed 12-week intervals, with one session each, and are therefore not included in the evaluation of the number of sessions.

Complications and side effects were rare in both groups. One patient (4.3%) with a fulminant allergic skin reaction with fever, itching and hives occurred in the PE group and one patient (4.8%) with mild hypotension in the IA group.

## Discussion

In this retrospective data analysis, efficacy and safety were demonstrated for both extracorporeal therapy procedures (IA and PE) in CIDP. It was shown that both therapy groups benefited from PE and IA and that more than half of the patients experienced an improvement in symptoms and signs. All patients had a long duration of disease and pre-treatments (e.g. corticosteroids and IVIg). Nevertheless, there was an improvement in INCAT and Hughes scores associated with IA and PE, and the patients benefited from these therapies even after a long period of illness and immunomodulatory pre-treatment. PE is recommended in the European Academy of Neurology/Peripheral Nerve Society (EAN/PNS) guidelines [6]. However in guidelines, there are no recommendations for IA due to insufficient study data. Short-term benefits can be expected with both procedures, but further deterioration has been described after both modalities [26]. In CIDP, treatment with PE is given a category I recommendation, level 1B [27]. For IA, there is no explicit mention in the ASFA guidelines that it is directly classified in the same category as PE, but IA can also be considered as a treatment option for CIDP, especially in treatment-refractory cases [10, 26]. 

IA involves selective elimination of autoantibodies and usually no reinfusion of substitutes (fresh frozen plasma (FFP), albumin), which offers theoretical advantages over PE. IA-treated patients generally do not require transfused proteins, which may increase the tolerability and safety of this treatment method with regard to serious complications [9, 11–14]. While we could not detect a systematic advantage of IA in our sample in this respect, the only serious allergic reaction did occur in the PA group. The length of stay and the number of apheresis sessions were the only parameters significantly different between treatment groups in this study; however, it could be explained by the larger standard deviation in IA group.

In this study, both therapies were safe and well tolerated and had almost no serve side effects, up to an allergic reaction in the PE group and mild hypotension in the IA group, comparable to other studies [7, 11]. In a prospective study, which only included 6 CIDP patients, the authors showed that IA was well tolerated and effective for longer term use, based on an improvement in INCAT and Hughes scores [19]. A prospective study of 20 patients with CIDP showed that IA is at least equally effective as PE based on INCAT and MRC scores as endpoints [20]. A retrospective study of 14 patients with CIDP also reported on the effectiveness and safety of IA in chronic immune neuropathies, but did not include PE patients [9]. Response to therapies was measured by improvements in strength, sensation and the ability to cope with daily tasks, and by changes in the INCAT score; any improvement was considered a treatment success [9, 20]. In the publications mentioned above, the patients had a shorter duration of disease and all received previous treatments. These patients were treated as a second-line therapy, and they showed an improvement [9, 19, 20]. In contrast, the patients in our study group had a longer duration of disease. They also received previous treatments and still benefited from the extracorporeal therapy procedures.

Limitations of this study include the retrospective data collection and, therefore, different adsorbers in IA and lack of a standardized follow-up regime. This makes it difficult to control for confounding factors (e.g. uniform collection of the INCAT and Hughes scores, local availability of one or the other apheresis method, variable intervals between treatment and clinical follow-up evaluation). Furthermore, both scores collected (INCAT, Hughes) are investigator dependent and there are more disease specific and sensitive instruments (e.g. MRC-SS or I-RODS) and their absence reduces the ability to capture subtle, disease-specific changes in functional status, thereby limiting the depth and specificity of outcome assessment in this study.

Testing for paranodal antibodies was not performed at the time of data acquisition, which limits our ability to assess their potential role in the patient’s neuropathological profile.

Despite the higher number of patients (*n* = 44) compared to previous studies, both the study design and the still small size of study population were not sufficient to make any statements on statistical significance.

In summary, the efficacy of IA is comparable to that of PE, and both are adequate treatment methods for chronic immune neuropathies. IA or PE as therapeutic escalation should be considered as a possible treatment option despite prolonged disease progression. It is particularly noteworthy that escalation of therapy in patients with CIDP under standard therapy leads to a significant improvement in the clinical condition. This clearly shows that there seems to be a therapeutic reserve in CIDP, which is underlined by the current studies using complement inhibition Fc receptor antibodies. Further studies must show whether earlier therapy escalation improves the long-term outcome of patients. Both extracorporeal therapy procedures improved INCAT and Hughes scores as well as the secondary outcome variables (number of sessions per therapy method and therapy success, measured by the subjective perception of patients and examiners). Thus, both modalities can be recommended, and decisions can be based on, e.g., local availability. A theoretical advantage of IA may be the shorter stay in hospital. However, further studies in a multicenter, and prospective design with a larger population are required to establish the efficacy of extracorporeal therapy procedures.

## Data Availability

The datasets during and/or analysed during the current study available from the corresponding author on reasonable request.
